# Diversifying the anthracycline class of anti-cancer drugs identifies aclarubicin for superior survival of acute myeloid leukemia patients

**DOI:** 10.1186/s12943-024-02034-7

**Published:** 2024-06-04

**Authors:** Xiaohang Qiao, Sabina Y. van der Zanden, Xiaoyang Li, Minkang Tan, Yunxiang Zhang, Ji-Ying Song, Merle A. van Gelder, Feija L. Hamoen, Lennert Janssen, Charlotte L. Zuur, Baoxu Pang, Olaf van Tellingen, Junmin Li, Jacques Neefjes

**Affiliations:** 1 Division of Tumor Biology and Immunology, The Netherlands Cancer Institute, Amsterdam, The Netherlands; 2 Department of Head and Neck Oncology and Surgery, The Netherlands Cancer Institute, Amsterdam, The Netherlands; 3 Department of Cell and Chemical Biology, ONCODE Institute, Leiden University Medical Center, Leiden, The Netherlands; 4 Shanghai Institute of Hematology, State Key Laboratory of Medical Genomics, National Research Center for Translational Medicine, Ruijin Hospital Affiliated to Shanghai Jiao Tong University School of Medicine, Shanghai, China; 5 Division of Pharmacology, The Netherlands Cancer Institute, Amsterdam, The Netherlands; 6 Division of Experimental Animal Pathology, The Netherlands Cancer Institute, Amsterdam, The Netherlands; 7 Wuxi Branch of Ruijin Hospital, Ruijin Hospital, Shanghai Jiao Tong University School of Medicine, Shanghai , 200025 China

**Keywords:** Anthracycline, Doxorubicin, Aclarubicin, Refractory/relapsed acute myeloid leukemia, Cardiotoxicity, Histone eviction, Chromatin damage, DNA damage, Bio-distribution, Cross-resistance

## Abstract

The efficacy of anthracycline-based chemotherapeutics, which include doxorubicin and its structural relatives daunorubicin and idarubicin, remains almost unmatched in oncology, despite a side effect profile including cumulative dose-dependent cardiotoxicity, therapy-related malignancies and infertility. Detoxifying anthracyclines while preserving their anti-neoplastic effects is arguably a major unmet need in modern oncology, as cardiovascular complications that limit anti-cancer treatment are a leading cause of morbidity and mortality among the 17 million cancer survivors in the U.S. In this study, we examined different clinically relevant anthracycline drugs for a series of features including mode of action (chromatin and DNA damage), bio-distribution, anti-tumor efficacy and cardiotoxicity in pre-clinical models and patients. The different anthracycline drugs have surprisingly individual efficacy and toxicity profiles. In particular, aclarubicin stands out in pre-clinical models and clinical studies, as it potently kills cancer cells, lacks cardiotoxicity, and can be safely administered even after the maximum cumulative dose of either doxorubicin or idarubicin has been reached. Retrospective analysis of aclarubicin used as second-line treatment for relapsed/refractory AML patients showed survival effects similar to its use in first line, leading to a notable 23% increase in 5-year overall survival compared to other intensive chemotherapies. Considering individual anthracyclines as distinct entities unveils new treatment options, such as the identification of aclarubicin, which significantly improves the survival outcomes of AML patients while mitigating the treatment-limiting side-effects. Building upon these findings, an international multicenter Phase III prospective study is prepared, to integrate aclarubicin into the treatment of relapsed/refractory AML patients.

**Supplementary Information:**

The online version contains supplementary material available at 10.1186/s12943-024-02034-7.

## Graphical abstract

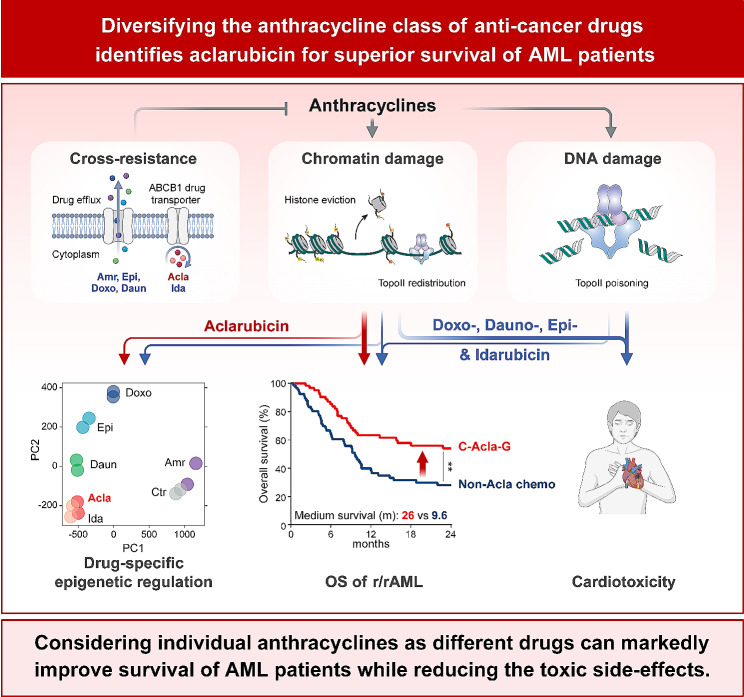

## Background

Most anthracyclines function as poisons of the enzyme topoisomerase II (TopoII) that trap it on chromatin in a state inducing DNA breaks [1]. In addition, these drugs also evict histones from defined genomic areas, leading to delay of DNA damage repair and alterations in epigenome and transcriptome, collectively termed chromatin damage [2–4]. Histone eviction emerges as a major cytotoxic mechanism of anthracyclines [5]. TopoII poisons, like etoposide, which solely induce DNA damage are considerably less effective in cancer treatment [2, 6], and also less toxic [5, 7]. On the contrary, aclarubicin that only induces histone eviction without causing DNA damage is highly effective in treating *de novo* acute myeloid leukemia (AML) [5, 8]. Hence, DNA damage appears not to be a prerequisite for effective anti-cancer activities of anthracycline drugs. We previously reported that the combination of DNA-damaging and chromatin-damaging activities leads to the cardiotoxicity and therapy-related tumorigenesis of anthracyclines [5]. Selectively eliminating the DNA-damaging activity of anthracycline from its chromatin-damaging activity through chemical modification mitigates these devastating side effects, without hampering their anti-cancer efficacies [5]. Cancer patients receiving anthracycline chemotherapy have more than twice the risk of developing heart failure compared to their peers without cancer [9, 10]. Moreover, this risk continues to increase with the survival time of cancer survivors [10]. Exceeding the recommended cumulative dose precipitates a sharp rise in cardiotoxicity [11]. Therefore, the cardiotoxicity of anthracyclines significantly limits patients from extending treatment and affects their quality of life. Clinical application of low cardiotoxic anthracyclines will confer major benefits in cancer therapy, expanding treatment options for patients with comorbidities including older adults [12] and reducing late effects, particularly for pediatric patients [13]. Low cardiotoxic anthracyclines would allow long-term treatment, while the current cardiotoxic anthracyclines limit treatment due to cumulative dose-dependent cardiotoxicity.

Here, we systematically evaluated anthracycline drugs currently used in clinic against a series of key features to determine whether they differ in terms of anti-neoplastic potency, site of action and toxicity profiles. We identified aclarubicin, an anthracycline currently used only in Asia for AML patients with high comorbidity indices, as a promising variant devoid of cardiotoxicity. Notably, aclarubicin can be safely administered even after maximum exposure to doxorubicin or idarubicin in mice and humans. When used in second line, aclarubicin-based regimen significantly increases the overall survival of relapsed/refractory AML patients by 23% compared to other intensive chemotherapy approaches. These findings illustrate how aclarubicin can improve the treatment of relapsed/refractory AML patients that currently have a poor prognosis, addressing a major unmet need in current oncology practice.

## Methods

### Reagents

Doxorubicin and etoposide were purchased from Pharmachemie (The Netherlands), daunorubicin was obtained from Sanofi-Aventis and epirubicin was obtained from Accord Healthcare Limited (UK), idarubicin was obtained from Pfizer and Santa Cruz Biotechnology (sc-204774). Aclarubicin for in vivo mouse experiments was obtained from Shenzhen Main Luck Pharmaceuticals Inc. Aclarubicin (for in vitro experiments, sc-200160) and amrubicin (sc-207289) were obtained from Santa Cruz Biotechnology.

### Cell culture

K562 (B. Pang, LUMC Leiden, The Netherlands, RRID: CVCL_K562), MM6 (RRID: CVCL_1426), MOLM13 (RRID: CVCL_1426), MV4:11 (RRID: CVCL_0064), U937 (RRID: CVCL_0007) (all four lines were a gift from L. Smit, VUMC Amsterdam, The Netherlands) and THP-1 (ATCC, Manassas, VA, RRID: CVCL_0006) were cultured in RPMI-1640 medium supplemented with 8% fetal calf serum (FCS, SERANA, S-FBS-CO-015). OCI-AML-2 (DSMZ, ACC-No 99, RRID: CVCL_1619) and OCI-AML-3 (DSMZ, ACC-No 582, RRID: CVCL_1844) (M. Griffioen, LUMC Leiden, The Netherlands) and OCI-AML-4 (M.L.M. Jongsma, LUMC Leiden, The Netherlands, RRID: CVCL_5224) were cultured in IMDM medium supplemented with 8% FCS and glutamine. MelJuSo (RRID: CVCL_1403) cells were maintained in IMDM supplemented with 8% FCS. All cell lines were maintained in a humidified atmosphere of 5% CO_2_ at 37 °C, regularly tested for the absence of mycoplasma and STR profile.

### Cell line construction

For endogenous tagged GFP-H2B K562 cells, mScarlet was swapped for GFP in the homology repair construct using NheI and BglII and cells were generated as described [5]. Co-transfection into K562 cells was done by electroporation using Lonza SF cell line kit. ABCB1 overexpressing K562 cells were generated as described [14]. Endogenous tagged 3×Flag-TopoIIα K562 cell line was generated using HR-3×Flag construct designed at least 40 base pairs up- and downstream of the genomic TopoIIα stop codon. The gRNA target sequence was designed using the Zhang Lab CRISPR tool (http://crispr.mit.edu/) and cloned into the pX330 vector (RRID: Addgene_110403). Primers used for the HR construct: 5’-CACCGATGATCTGTTTTAAAATGTG-3’ and 5’-AAACCACATTTTAAAACAGATCATC-3’. Co-transfection of ssDNA oligo and CRISPR plasmid (pX459) into K562 cells was performed by electroporation using Lonza SF cell line kit. Primers used for genotyping were forward primer: 5’-TAAGCAGAATTCATGCCACTTATTTGGGCAAT-3’ and reverse primer: 5’-TGCTTAAAGCTTTGCCCATGAGATGGTCACTA-3’.

### DNA damage assessed by Western blot and constant field gel electrophoresis

After 2-hour drug treatment of indicated drugs at 5 µM, THP-1 cells were washed with PBS, and then lysed directly in sodium dodecyl sulfate (SDS)-sample buffer (2% SDS, 10% glycerol, 5% β-mercaptoethanol, 60 mM Tris-HCl pH 6.8, and 0.01% bromophenol blue). Lysates were resolved by SDS/polyacrylamide gel electrophoresis followed by Western blotting. Primary antibodies used for blotting were γH2AX (1:1,000, 05-036, Millipore) and β-actin (1:10,000, A5441, Sigma, RRID: AB_476744). CFGE were performed as described [15]. Images were quantified with ImageJ (RRID: SCR_003070).

### Fractionation assay

Endogenously tagged GFP-H2B K562 cells (1 × 10^6^) were pre-incubated with protease inhibitor cocktail (P1860-1ML, Sigma-Aldrich) for 1 h followed by 3-hour treatment with 10 µM of the indicated drugs. Cells were dissolved in lysis buffer (50 mM Tris-HCl pH 8.0, 150 mM NaCl, 5 mM MgCl_2_, 0.5% NP40, 2.5% glycerol supplemented with protease inhibitors and 10 mM NMM) for 5 min on ice, and then centrifuged for 10 min at 15,000 g at 4 °C. Both nucleus (pellet) and cytosol (supernatant) were washed once and then submitted for Western Blot analysis. Primary antibodies used for detection: GFP (1:1,000) [16], Lamin B1 (1:1,000, 12,987-I-AP, Proteintech, RRID: AB_2136290), and Calnexin (1:1,000, #2679, Cell Signaling Technology, RRID: AB_2228381).

### Time-lapse confocal microscopy

For time-lapse confocal imaging, MelJuSo cells were seeded in 35-mm glass bottom petri dish (Poly-D-Lysine coated, MatTek Corporation), transfected with TopoIIα-GFP construct using Effectene (301425, QIAGEN) and imaged upon treatment with the indicated drugs [2]. Leica SP8 confocal microscope system, 63× lens, equipped with a climate chamber was used. TopoIIα-GFP distribution was quantified using Leica Application Suite X software (RRID: SCR_013673).

### Short-term cell viability assay

Twenty-four hours after seeding into 96-well plates, cells were treated with indicated drugs for 2 h at physiologically relevant concentrations [2]. Subsequently, drugs were removed by extensive washing, and cells were cultured for an additional 72 h, according to their pharmacokinetics. Cell viability was measured using the CellTiter-Blue viability assay (G8080, Promega). Survival was normalized to the untreated control samples after correction for the background signal.

### ChIP-seq

Endogenous tagged 3×Flag-TopoIIα K562 cells were treated with 10 µM of indicated drugs for 4 h. Cells were fixed and processed as described [3, 17]. ChIP was done with anti-Flag M2 antibody (F3165, Sigma, RRID: AB_259529), followed by sequencing on an Illumina Hiseq2000 platform (Genome Sequencing Service Center of Stanford Center for Genomics and Personalized Medicine Sequencing Center).

ChIP-seq data were processed identically using the ENCODE Data Coordination Center (DCC) ChIP-seq pipeline (https://github.com/ENCODE-DCC/chip-seq-pipeline2) (v1.9.0). Briefly, the ChIP-seq reads were aligned to the human reference genome (GRCh37/hg19) using Bowtie2 (RRID: SCR_016368) [18]. Duplicate reads were removed using Picard MarkDuplicates (RRID: SCR_006525). Peaks of each sample were called against the whole-cell lysate replicates using SPP (RRID: SCR_001790) [19] with the parameters ‘-npeak 300000 -speak 155 -fdr 0.01’. The blacklisted regions described by ENCODE were discarded [20]. Reproducible peaks were intersected from two biological replicates and annotated with epigenomic signatures of K562 cells, downloaded from the Roadmap Epigenomics Project (RRID: SCR_008924) [21]. Normalized TopoIIα binding affinity matrix: consensus peaks by samples, principle component analysis and differential binding affinity analysis were performed using the R package DiffBind (RRID: SCR_012918) [22].

### ATAC-seq

Wild-type K562 cells were treated with 10 µM of indicated drugs for 4 h. Cells were fixed and processed as described [23, 24]. DNA was processed using a customized library preparation method for ATAC-seq and was sequenced using an Illumina HiSeq4000 platform.

ATAC-seq data were processed identically using the ENCODE Data Coordination Center (DCC) ATAC-seq pipeline (https://github.com/ENCODE-DCC/atac-seq-pipeline) (v1.10.0). Briefly, the ATAC-seq reads were aligned to the human reference genome (GRCh37/hg19) using Bowtie2 [18]. Duplicate reads were removed using Picard MarkDuplicates (RRID: SCR_006525). Peaks were called using MACS2 (RRID: SCR_013291) [25] with the setting of ‘-p 0.01 --shift − 75 --extsize 150 --nomodel -B --SPMR --keep-dup all’, then followed by blacklisted regions filtering described by ENCODE [20]. Reproducible peaks were identified from two biological replicates and annotated with epigenomic signatures of K562, downloaded from the Roadmap Epigenomics Project [21]. ATAC-seq signal tracks for all the samples were generated by BEDTools (RRID: SCR_006646) with the command ‘bedtools genomecov -scale’ using the read count per million (CPM) normalization and convert to bigwig files using bedGraphToBigWig. Normalized ATAC-seq read density matrix: consensus peaks by samples, principle component analysis and differential chromatin accessibility analysis were performed using the R package DiffBind [22].

### Principal component analysis (PCA)

The reproducible peaks were identified by intersecting two biological replicates of each drug treatment, and then merged into consensus peak set. Subsequently, the read density was quantified for the consensus peaks to create an input matrix for PCA analysis. The dba.plotPCA function of DiffBind package was applied to calculate the principal components through eigenvector decomposition of the covariance matrix from the input matrix. The maximum variance of projected data by principal component 1 and 2 were plotted in the x- and y-axis accordingly.

### DNA dye exclusion assay

Circular DNA (1 µg/ml) was incubated with Quant-iT PicoGreen dsDNA reagent (P7581, Thermo Fisher Scientific) for 5 min at room temperature (RT). Subsequently, drug was added to the DNA/PicoGreen mix at indicated concentrations and incubated for another 5 min at RT. Following the reaction, the PicoGreen fluorescence was measured using CLARIOstar plate reader (BMG labtech) with excitation at 480 nm and emission at 520 nm (48020/52010 filter). The fluorescence was quantified relative to untreated controls. Fluorescent signals of all samples were corrected for the corresponding drug concentrations in the absence of DNA.

### Bio-distribution of anthracyclines in mice

FVB/NRj mice (RRID: MGI:6364162) ordered from Janvier Labs (Le Genest-Saint-Isle, France) were housed in individually ventilated cages under specific pathogen-free conditions in the animal facility of the NKI (Amsterdam, The Netherlands). All mouse experiments were approved by the Animal Ethics Committee of the NKI and were performed according to institutional and national guidelines. Male mice (8-week old) were i.v. injected with doxorubicin, aclarubicin, amrubicin, epirubicin or idarubicin at 5 mg/kg (*n* = 5 per group). Four hours post injection, animals were sacrificed, and plasma, heart, lung, liver, kidney, spleen, brain, thymus, axillary + inguinal lymph nodes, and testis + epididymis were collected. Hearts were cut into two pieces with coronal section. One piece was fixed in EAF fixative (ethanol/acetic acid/formaldehyde/saline, 40:5:10:45 v/v/v/v) and processed for Phospho-H2AX (Ser139) IHC (1:100, #2577, Cell Signaling Technology). The other half of the heart and the rest of organs were weighed and frozen.

Calibration samples for each anthracycline with defined concentration were prepared in blank tissue homogenates. Daunorubicin was used as internal standards. Of each sample, a 100 µl tissue homogenate was mixed with 200 µl of 0.1% formic acid, 1 ml of Chloroform:2-propanol (1:1) and 10 µl of internal standard, then vortexed vigorously for 5 min, followed by centrifugation at 5,000 g for 5 min at 4 °C. The aqueous layer and the intermediate layer were removed by suction. The organic layer was decanted into a clean polypropylene tube and evaporated by Savant Speed-Vac SC210A concentrator (Thermo Fisher Scientific, Waltham, USA) at 35 °C. The residue was reconstituted in 150 µl of DMSO, vortexed for 20 sec and then sonicated for 5 min. After centrifuged at > 12,000 g for 2 min, 10 µl of supernatant was analyzed by liquid chromatography-tandem mass spectrometry (LC-MS)/MS, which consisted of an API 3500 mass spectrometer (Sciex, Framingham, MA) coupled to an UltiMate 3000 LC System (Dionex, Sunnyvale, CA). Samples were separated using a ZORBAX Extend-C18 column (Agilent, Santa Clara, CA), kept at 50 °C preceded by a Securityguard C18 pre-column (Phenomenex, Utrecht, The Netherlands). Elution was done using a mixture of mobile phase A (0.1% formic acid in water (v/v)) and mobile phase B (methanol) in a 2 min gradient from 20 to 95% B, followed by 95% B that was maintained for 3 min and then re-equilibrated at 20% B. Multiple reaction monitoring parameters were 544.0/397.0 (doxorubicin and epirubicin), 498.1/291 (idarubicin), 812.4/570.3 (aclarubicin), 484.2/333.1 (amrubicin) and 528.1/321.1 (daunorubicin). System control and data analysis were done using Analyst® 1.6.2 software (AB Sciex; Foster City, CA).

### The cardiotoxicity of anthracyclines in mice

FVB/NRj mice (10–11-week old, RRID: MGI:6364162) were i.v. injected with 5 mg/kg of doxorubicin, 5 mg/kg of aclarubicin, or 5 ml/kg of saline every 2 weeks for 4 times. After 4-week interval, the animals were i.v. injected with 5 mg/kg of indicated drug or 5 ml/kg of saline every 2 weeks for another 4 times. The mice were monitored every other day. When body weight loss was more than 20%, or circulation failure occurred, animals were euthanized by CO_2_. Subsequently, full body anatomy was performed. All organs were collected, fixed in EAF fixative and embedded in paraffin. Sections were cut at 2 μm from the paraffin blocks and stained with hematoxylin and eosin, and 4 μm for immunohistochemistry of Desmin (1:200, M0760, DakoCytomation), Vimentin (1:100, #5741, Cell Signaling Technology, RRID: AB_10695459), or Periostin (1:100, ab215199, Abcam, RRID: AB_2924310). Pathology slides were reviewed twice by an expert mouse pathologist who was blind to the treatment. Incidence rate (IR = [number of mice with the specific side effect over a time period]/[sum of mice × time at risk during the same time period]) and cumulative incidence (CI = [number of mice with specific side effect at end time point]/[total number of mice at start]) were calculated for cardiotoxicity.

### AML patient data analyses

Patients with refractory or relapsed AML treated between July 2012 and July 2022 at Ruijin hospital, China were enrolled in this retrospective study. Some patients participated in trial ChiCTR-OPC-14,005,712, ChiCTR-OPC-15,006,896, ChiCTR-IIR-16,008,809, ChiCTR-OIC-16,008,952, ChiCTR-IIR-16,008,962 and ChiCTR-IIR-17,011,677. This study was approved by the ethics committee of Ruijin Hospital, all patients provided written informed consent.

Cytogenetic risk was classified according to the modified Southwest Oncology Group criteria [26]: (1) favorable risk, including t(8;21) and inv(16) or t(16;16)(p13;q22); (2) unfavorable risk, including del(5q) or monosomy 5, monosomy 7 or del(7q), abnormal 3q, 9q, 11q, 21q, or 17p, t(6;9), t(9;22), and complex karyotypes (three or more unrelated chromosomes abnormal); and (3) intermediate risk, including normal karyotypes and all other anomalies. FLT3 internal tandem duplication (FLT3-ITD) and mutations in CEBPA, NPM1 and IDH1/2 were tested. Integrated risk was classified as described [27]. Complete remission was defined as bone marrow blasts < 5%, absolute neutrophil count ≥ 1 × 10^9^/L, and platelet count ≥ 100 × 10^9^/L, and absence of extramedullary disease. Partial remission was defined as having < 15% (and a 50% decrease in bone marrow blasts) but > 5% blasts or with < 5% blasts but not reaching the CR criteria for blood cell count or clinical manifestation. The baseline characteristics and clinical outcomes of the patients are summarized in supplemental Tables S1 and S2, respectively.

### AML treatments

CAG patients were treated with 15‒25 mg/m^2^ of cytarabine (Ara-C) injected s.c. every 12 h on days 1‒14, 20 mg/day of aclarubicin infused i.v. on days 1‒4, and 200 µg/m^2^ of granulocyte stimulating factor (G-CSF) administered s.c. daily on days 1‒14. G-CSF was reduced, or temporarily stopped when neutrophilia was > 5 × 10^9^/L. IA patients were treated with 6‒10 mg/m^2^ of idarubicin infused i.v. on days 1‒3 and 100‒200 mg/m^2^ of Ara-C on days 1‒7. VA patients were injected with 75 mg/m^2^ of azacitidine s.c. daily on days 1‒7, and administered with venetoclax orally, once daily. The dose of venetoclax was 100 mg on day 1 and 200 mg on day 2; and 400 mg on days 3‒28. In all subsequent 28-day cycles, the dose of venetoclax was initiated at 400 mg daily. The other induction chemotherapies for r/rAML patients included IA, DA, FLAG, CLAAG and CHA regimens. For patients treated with DA regimen, 20 mg/m^2^ of decitabine was administered i.v. daily on days 1‒5, and 1 g/m^2^ of Ara-C was injected every 12 h on days 6‒7. For patients treated with FLAG regimen, fludarabine was infused i.v. at 30 mg/m^2^ on days 2‒6; 4 h after fludarabine infusion, Ara-C was injected i.v. at 1.5–2 g/m^2^ over 3 h on days 2‒6; G-CSF was administered at 5 µg/kg s.c. on days 1‒5; additional G-CSF may be administered since 7 days after the end of chemotherapy until white blood cell count > 500/μL. For patients > 60-year-old, the dose may be reduced to 20 mg/m^2^ for fludarabine and 0.5–1 g/m^2^ for cytarabine. For patients treated with CLAAG regimen, 5 mg/m^2^ of cladribine was infused i.v. over 2 hours on days 1‒5; 15 mg/m^2^ of Ara-C was injected s.c. every 12 h on days 1‒10; trans retinoic acid (ATRA) was administered orally at 45 mg/m^2^ on days 4‒6, then at 15 mg/m^2^ on days 7‒20; 300 µg G-CSF was injected s.c. on day 0. For patients treated with CHA regimen, 5 mg/m^2^ of cladribine was infused i.v. over 2 hours on days 1‒5; 2 mg/m^2^ of homoharringtonine was infused i.v. over 2 hours on days 1‒5; 1 g/m^2^ of Ara-C was injected 2 hours after cladribine on days 1‒5.

### Statistical analyses

Results are shown as mean ± SEM or mean ± SD. Statistical analysis was performed using GraphPad Prism (RRID: SCR_002798) unless otherwise specified. All in vitro experiments were performed with a minimum of three independent trials with the exception of ChIP-seq and ATAC-seq which were biological duplicates. All individual animals in mouse experiments are shown in the dot plots. Statistical tests are indicated in each figure legend. Survival curves were analyzed by the log-rank (Mantel-Cox) test. Statistical analysis of pathology quantification was determined using the Student’s *t*-test or Mann-Whitney test. Kinetic analysis was performed with Two-way ANOVA. Clinical outcomes were analyzed by Fisher’s exact test or Mantel-Haenszel test. *P* values of < 0.05 were considered statistically significant.

## Results

### Mechanisms of action and cross-resistance of anthracyclines

**Fig. 1:**
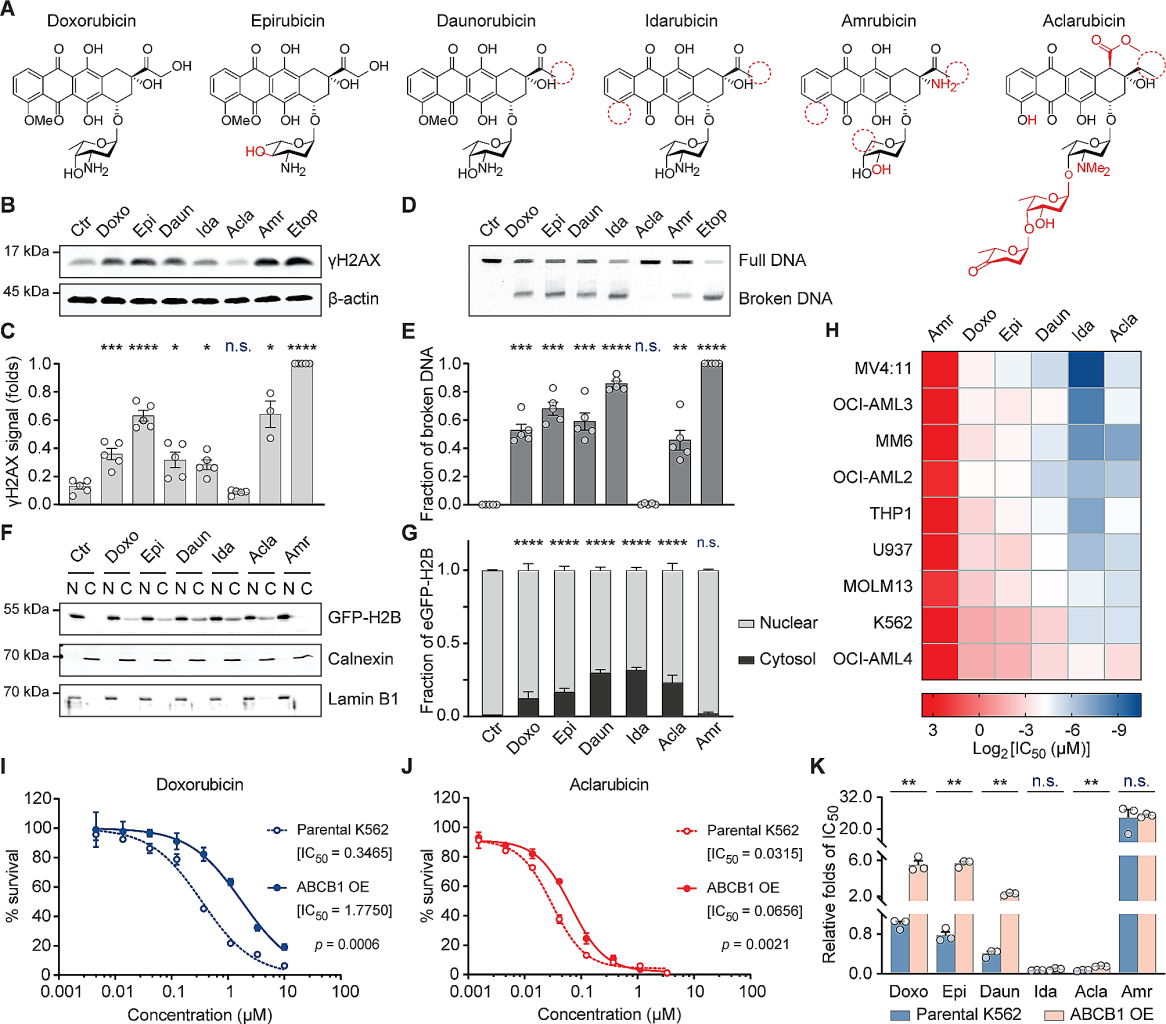
Acla differs from other anthracyclines in mechanisms of action and cross-resistance. (**A**) Structures of anthracyclines used in this study. Chemical features divergent from Doxo are depicted in red. (**B**) DNA damage examined by γH2AX Western blot in THP-1 cells. (**C**) Quantification of the γH2AX signal normalized to β-actin. Data are mean ± SEM; *n* = 4 biological replicates; Student’s *t*-test. (D) DSBs analyzed by CFGE in THP-1 cells. (**E**) Quantification of relative broken DNA in (**D**). Data are mean ± SEM; *n* = 4 biological replicates; Student’s *t*-test. (**F**) Histone eviction revealed by cell fractionation assay in K562-eGFP-H2B cells. N, nuclear fraction; C, cytosolic fraction. Calnexin and Lamin B1 are the loading control of each fraction. (**G**) The distribution of eGFP-H2B was quantified for both compartments. Data are mean ± SD; *n* = 4 biological replicates; two-way ANOVA. (**H**) IC_50_ of each anthracycline in different leukemia cell lines. (**I, J**) Cell viability of parental K562 cells and ABCB1-overexpressing K562 cells upon Doxo (**I**) and Acla (**J**) treatment. Data are mean ± SD; *n* = 3 biological replicates; two-way ANOVA. (**K**) Relative IC_50_ folds of each condition compared to that of Doxo in parental K562 cells. Data are mean ± SEM; *n* = 3 biological replicates; Student’s *t*-test. *P < 0.05, **P < 0.01, ***P < 0.001, and ****P < 0.0001; n.s., not significant

To assess whether the clinically administered anthracyclines (Fig. 1A) differ in their DNA- and chromatin-damaging activities, as well as cross-resistance, we exposed an AML cell line THP-1 to clinically relevant doses for 2 h [28]. DNA damage was evaluated using constant-field gel electrophoresis (CFGE) [15] and detection of phosphorylation of H2AX at Ser139 (γH2AX) [29] by Western blotting. The anthracyclines, doxorubicin (Doxo), daunorubicin (Daun), epirubicin (Epi), idarubicin (Ida), amrubicin (Amr) and structure-unrelated TopoII poison etoposide (Etop) all induced DSBs, unlike aclarubicin (Acla) (Fig. 1B–E). Next, chromatin damage as the result of histone eviction was visualized using fractionation assay in K562 cells with endogenously tagged GFP-H2B. Except for Amr, all the tested anthracyclines induced histone release from nucleus and accumulation in cytosol (Fig. 1F, G; Fig. S1A). Furthermore, histone-evicting anthracyclines also redistributed GFP-TopoIIα on chromatin (Fig. S1B, C). Cytotoxicity assays revealed poor anti-cancer activity for the DNA-damaging analog Amr, while the other analogs bearing chromatin-damaging activity were effective in eliminating various myeloid leukemia cell lines (Fig. 1H).

In addition to treatment-limiting cardiotoxicity, drug resistance could also limit the effect of anthracycline drugs. ABCB1, a major drug efflux transporter, contributes to anthracycline resistance [30, 31]. We generated a Doxo-resistant leukemia cell line by overexpressing ABCB1 [14] and tested its sensitivity to other anthracyclines (Fig. 1I–K; Fig. S1D). The ABCB1-overexpressing cells acquired resistance to Doxo, Daun, Epi and Amr, but failed to export Ida and Acla efficiently (Fig. 1J, K). These findings suggest that clinically relevant anthracyclines should be considered as distinct drugs with unique features and varying efficiencies. Evicting histones and relocalizing TopoIIα while not inducing detectable DNA damage, as in the case of Acla, appears adequate for efficient cytotoxicity.

### Epigenetic selectivity of TopoIIα redistribution and histone eviction of anthracyclines

Anthracyclines poison TopoII by disrupting its interface with DNA and trapping TopoII prior or after DNA damage formation. The cyclohexene ring ensures DNA intercalation while the sugar moiety fills the minor groove and attacks TopoII [32]. However, the anthracycline-specific redistribution of TopoII and its association with histone eviction have not been characterized. We addressed this in K562 cells, whose epigenomic landscape has been extensively profiled by the ENCODE consortium [33]. Chromatin immunoprecipitation followed by deep sequencing (ChIP-seq) against endogenously tagged TopoIIα was performed 4 h post anthracycline exposure in two independent experiments (Fig. S2A). Although anthracyclines can be sufficiently washed out from samples, trace amount of drugs might remain DNA-bound due to their intercalating properties [34]. The distribution pattern of total reads for anthracyclines did not align with that of DNA intercalating activity, suggesting that any residual anthracycline in the DNA did not significantly interfere with the subsequent sequencing steps (Supplementary Excel file; Fig. S2B).

**Fig. 2:**
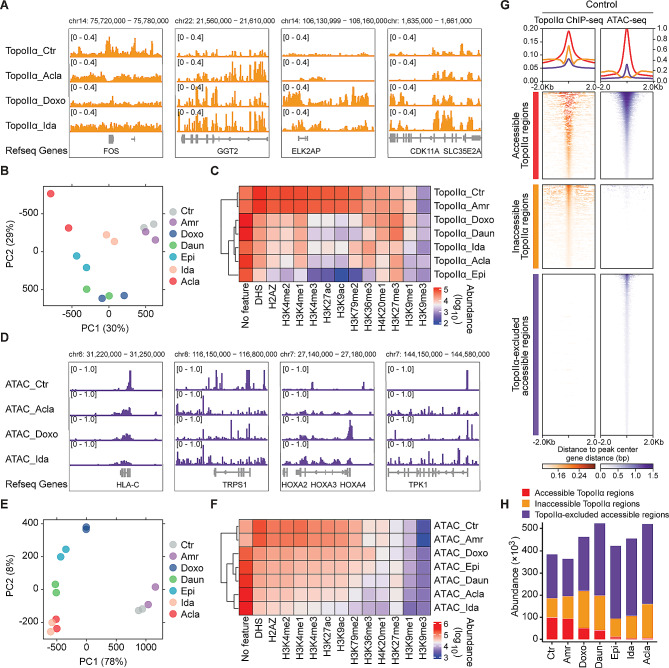
Epigenetic selectivity of TopoIIα redistribution and histone eviction of anthracyclines. (**A**) Illustration of drug-specific TopoIIα redistribution revealed by ChIP-seq. (**B**) Principal component analysis (PCA) of TopoIIα ChIP-seq data. Two independent biological replicates were included for each condition. (**C**) Heatmap of TopoIIα peak abundance associated with specific histone features derived from Roadmap Epigenomics Project. (**D**) Illustration of drug-specific accessible chromatin regions revealed by ATAC-seq. (**E**) PCA analysis of ATAC-seq data. Two independent biological replicates were included for each condition. (**F**) Heatmap of ATAC peak abundance associated with specific histone. (**G**) Density plots showing the accessible and inaccessible TopoIIα regions, and TopoIIα-excluded accessible regions surrounding ± 2 kb from the center of the detected peaks in the untreated K562 cells. (**H**) The abundance of each category in different conditions

Principal component analysis (PCA) of the resulting ChIP-seq profiles suggested that all histone-evicting anthracyclines cause extensive and drug-specific TopoIIα redistribution compared to untreated or Amr-treated cells (Fig. 2A, B). To characterize this drug-specific TopoIIα redistribution, we integrated the ChIP-seq data with the epigenetic information from ENCODE (Fig. 2C). Anthracyclines with the similar sugar moiety and cyclohexane (Fig. 1A), such as Daun and Doxo (also named as hydroxyDaun), depleted TopoIIα from active chromatin regions, including DNase I hypersensitive regions (DHS), H3K4me1-, H3K4me2-, H3K4me3-, H3K9ac-, H3K27ac-, H3K79me2- and H2A.Z-associated regions [35]. Instead, TopoIIα was trapped in compact chromatin regions marked by H3K27me3 [35], H3K9me1 [36] and H3K9me3 [37] (Fig. 2C). Ida and Acla depleted TopoIIα from broader chromatin states, except for H3K9me3- [37], H3K27me3- [35] and H3K36me3-decorated regions [37] (Fig. 2C). Epi, with an epimerizing hydroxyl group at the sugar moiety (Fig. 1A), further depleted TopoIIα from H3K36me3-modified regions [37] (Fig. 2C). It is worth noting that the redistribution of TopoIIα at regions with unknown epigenetic features was increased for all drugs, except for Amr (Fig. 2C).

While the TopoIIα redistribution exhibited drug specificity, we further investigated the histone eviction preferences of each anthracycline using transposase-accessible chromatin with sequencing (ATAC-seq) [23] in wild-type K562 cells (Fig. 2D). In line with its lack of chromatin-damaging activity (Fig. 1F, G), Amr failed to induce *de novo* open chromatin and clustered closely with untreated cells in PCA plot (Fig. 2E). Similar to the pattern of TopoIIα redistribution, the other tested anthracyclines predominantly evicted histones from open chromatin regions, highlighting their distinct preferences (Fig. 2F). The integration of TopoIIα ChIP-seq and ATAC-seq revealed a connection between histone eviction and TopoIIα depletion in transcriptionally active regions, suggesting that selective histone eviction may contribute to the observed depletion of TopoIIα in these regions (Fig. 2G, H; Fig. S2C). However, the presence of a significant number of non-overlapping regions between TopoIIα redistribution and *de novo* accessible chromatin suggests that they may constitute two independent effects of anthracyclines (Fig. S2C). The individual genomic preferences of clinically used anthracyclines imply that they should be considered as distinct drugs.

### Bio-distribution of anthracyclines

**Fig. 3:**
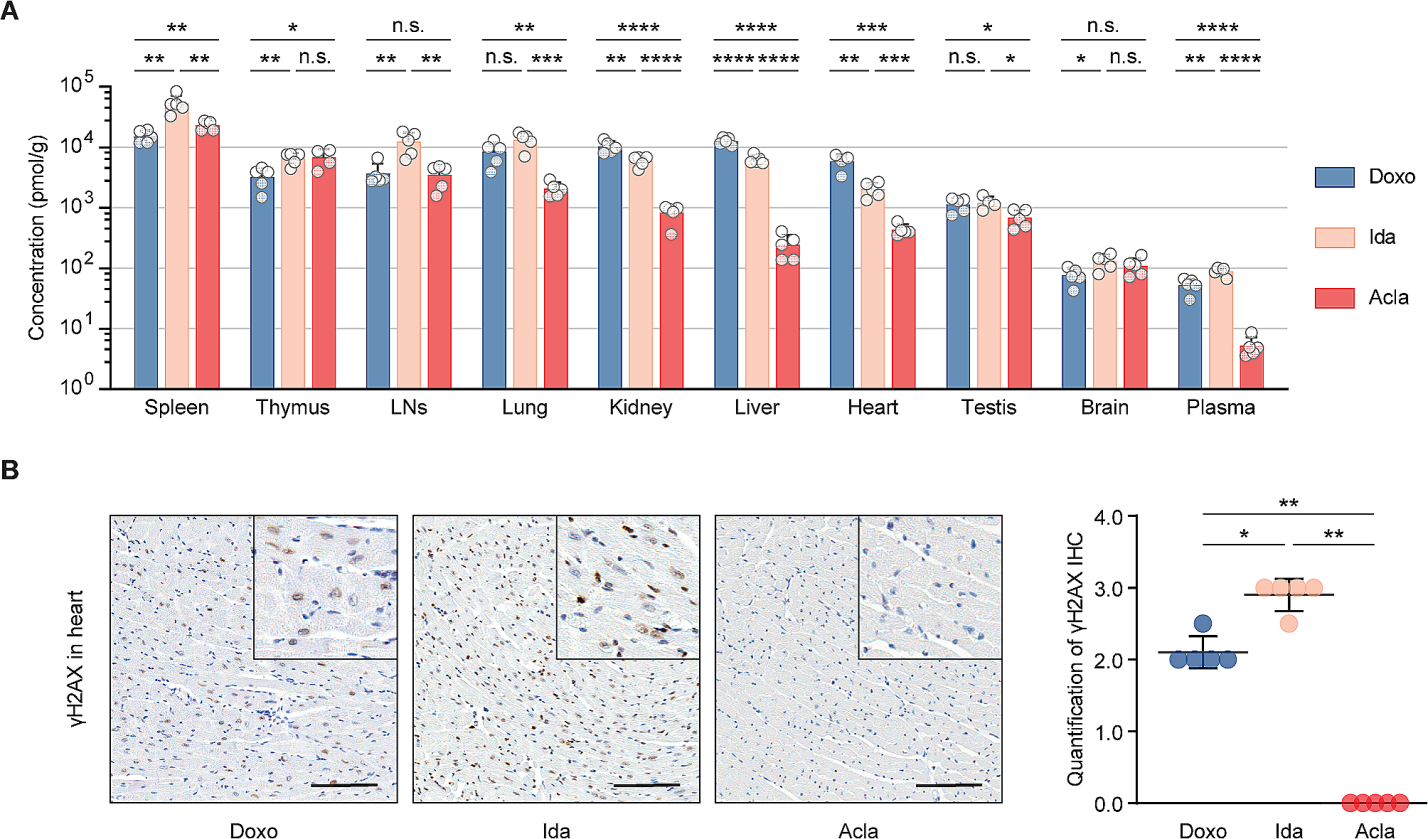
Bio-distribution of clinically used anthracyclines in mice. (**A**) Drug bio-distribution was determined 4 h after i.v. injection of indicated drug at 5 mg/kg. Data are represented as mean ± SD from 5 mice per group. Student’s *t*-test. (**B**) Representative microscopic images of γH2AX IHC staining of the hearts. Scale bars, 100 μm. Quantification is represented as mean ± SD, *n* = 5, Mann-Whitney test. *P < 0.05, **P < 0.01, ***P < 0.001, and ****P < 0.0001; n.s., not significant

Acla is effective in eliminating a large variety of cancer cells in tissue culture [5]. However, its clinical use is primarily confined to AML treatment, showing less efficacy against solid tumors [38, 39]. This discrepancy may stem from differences in biodistribution of anthracyclines. To test this, we performed a comprehensive bio-distribution study comparing Acla with the other clinically used anthracyclines in mice. Four hours post i.v. injection of a clinically relevant dose (5 mg/kg) [40] of indicated drug, Doxo, Epi and Ida exhibited similar bio-distribution patterns across different organs (Fig. 3A; Fig. S3A). Although Amr had a similar tissue distribution as Doxo, it showed reduced accumulation in lungs, kidneys, and heart. A notable exception was Acla, which accumulated in lymphoid organs (spleen, thymus and lymph nodes) but poorly distributed to other tissues (Fig. 3A; Fig. S3A). Acla rapidly decreased in plasma (Fig. 3A; Fig. S3A), mirroring observations in humans, mainly due to metabolism [41]. This distinct distribution pattern may explain the limited efficacy of Acla in treating solid tumors, as opposed to hematologic tumors. Brain tissue was poorly penetrated by all drugs (Fig. 3A; Fig. S3A).

Furthermore, we evaluated DNA damage in the heart following drug exposure. While Doxo, Epi and Ida induced persistent DNA damage in the heart, the hearts of Acla- and Amr-treated mice did not show any γH2AX signals 4 h post drug administration (Fig. 3B; Fig. S3B). This observation aligns with the poor tissue penetration of Acla and Amr, and the fact that Acla does not induce detectable DNA damage.

### Acla is safe and well tolerated following Doxo treatment

**Fig. 4:**
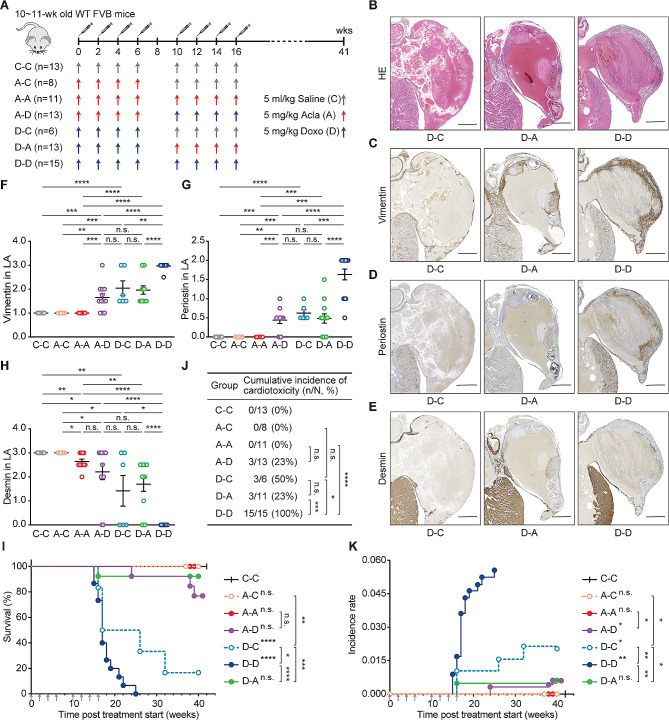
Acla is safe and well tolerated following Doxo treatment. (**A**) Wild-type FVB mice were i.v. injected with Doxo (**D**), Acla (**A**), or saline (**C**) as indicated. (**B-E**) Representative microscopic images of the left atrium (LA) of heart. Lesions caused by Doxo treatment represent as impairment of the wall, thrombosis, inflammation, fibrosis/calcification, and disappearing of the lumen of atrium (filled by a large thrombus). Scale bars, 500 μm. (**F-H**) Quantification of the indicated IHC staining in LA. Data are mean ± SEM, Mann-Whitney test. (**I**) Animal survival is plotted in Kaplan-Meier curves. Log-rank test. (**J**) Cumulative incidence of cardiotoxicity. Fisher’s exact test. (**K**) Incidence rate of cardiotoxicity. Two-way ANOVA with repeated measures, two-sided. *P < 0.05, **P < 0.01, ***P < 0.001, and ****P < 0.0001; n.s., not significant

Anthracyclines like Doxo, Daun, Ida and Epi are known to induce cumulative dose-dependent cardiotoxicity, often restricting patient treatment to a limited number of courses. Given that Acla treatment does not entail cardiotoxicity risk [5], we reasoned that it might be safe to administer this drug to mice following the treatment of cardiotoxic anthracyclines. To test this, mice received Acla injection after half-maximum cumulative dose of Doxo (Fig. 4A) and were monitored over time.

Doxo-induced cardiotoxicity resulted in cardiac hypertrophy and remodeling, evident from thrombus formation in the left atrium and auricle, accompanied by inflammation, fibrosis and calcification [5, 42] (Fig. 4B; Fig. S4A). Further staining for profibrotic proteins vimentin [43] and periostin [44], and cytoskeletal protein desmin [45], revealed myocyte impairment and stromal fibrosis (Fig. 4C–H; Fig. S4B–F). Ultimately, Doxo-treated mice died from circulation failure due to impaired cardiac function (Fig. 4I).

Consistent with clinical observations [9] and our previous findings [5], the incidence, latency and histopathological alterations of Doxo-induced cardiotoxicity manifested in a dose-dependent manner (Fig. 4, D-C vs. D-D). In contrast, eight courses of Acla treatment did not elicit any abnormalities in the heart (Fig. 4F–K; Fig. S4, A-A vs. C-C/D-C/D-D). More importantly, Acla treatment after half-maximum cumulative dose of Doxo did not aggravate cardiotoxicity (Fig. 4; Fig. S4, D-A vs. D-D). Likewise, pre-treatment of Acla did not render mice more susceptible to Doxo-induced cardiotoxicity (Fig. 4; Fig. S4, A-D vs. D-C/D-D). Hence, Acla is safe and well tolerated following Doxo treatment in this model system. Considering its limited cardiotoxicity, minimal cross-resistance and distinct epigenomic specificities compared to other anthracyclines, Acla may act as an independent therapeutic agent in salvage relapsed/refractory AML (r/rAML) patients who have reached the maximum cumulative dose of cardiotoxic anthracyclines. However, systematic studies examining this aspect are lacking.

### A combination therapy of cytarabine, aclarubicin and G-CSF (CAG) significantly improves the survival of r/rAML patients

r/rAML poses a significantly challenge in hematology, with a 5-year overall survival (OS) of only 10% [46]. The prognosis of elderly or unfit r/rAML patients is even more disappointing [47]. Unfortunately, no specific salvage regimen has emerged as a standard treatment for r/rAML [47]. The CAG regimen, containing low-dose cytarabine, Acla and G-CSF, has been widely used in China and Japan for treating AML [48, 49]. It is well tolerated by relapsed/refractory and elderly AML patients with lower toxicity than conventional chemotherapies [48–50]. To compare the efficacy of CAG to other salvage chemotherapies in r/rAML, we conducted a single-center retrospective study at Ruijin hospital (Shanghai, China).

**Fig. 5:**
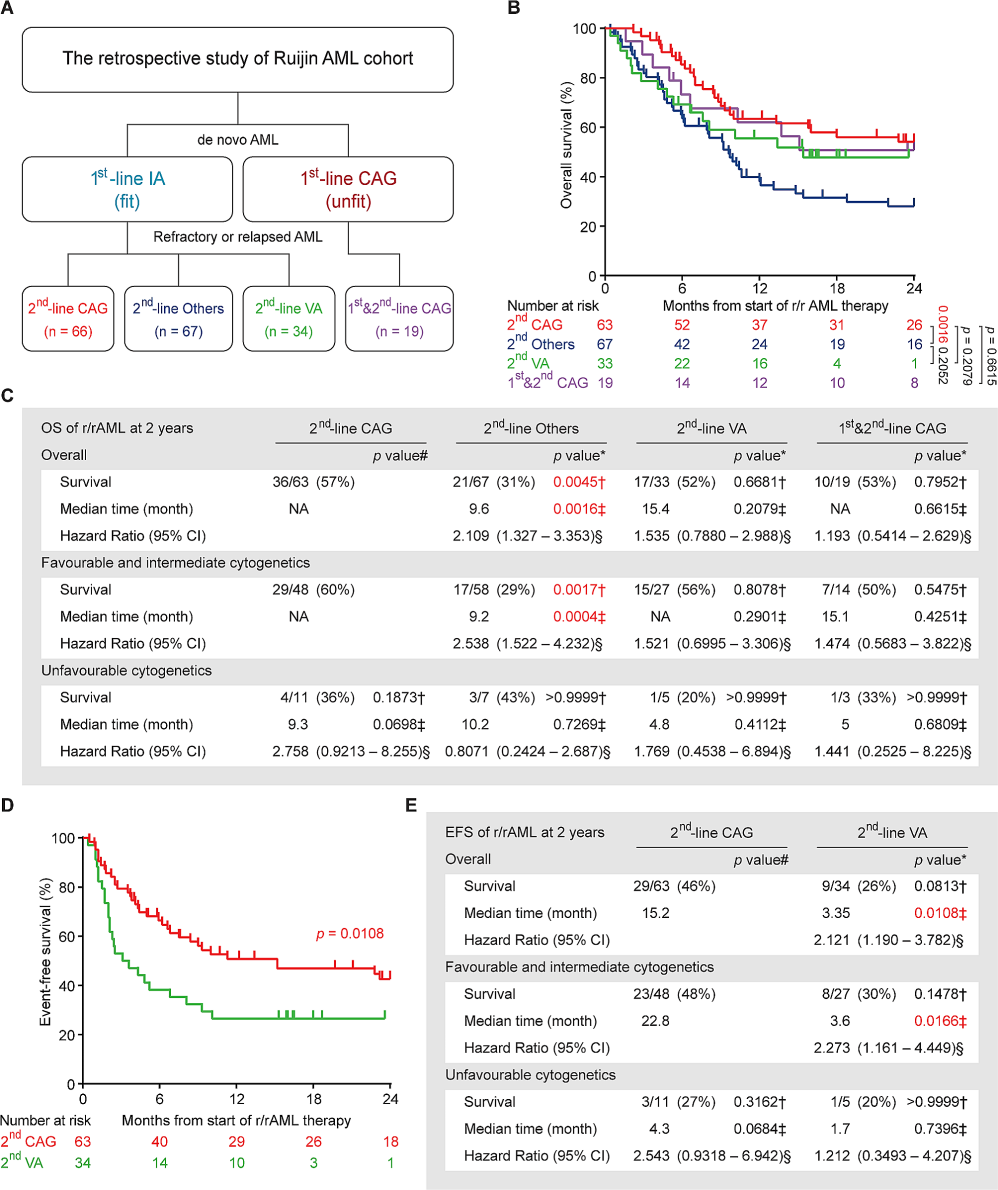
CAG is effective in treating r/rAML patients. (**A**) The therapy overview of Ruijin AML cohort. CAG: cytarabin, aclarubicin, G-CSF; IA: idarubicin, cytarabine; VA: venetoclax, azacitidine; Others: chemotherapy regimens other than CAG. (**B**) Overall survival of the r/rAML patients from the start of r/rAML induction treatment to the date of death. Log-rank test. (**C**) The statistical analysis of OS of r/rAML patients at 2 years. Data are n/N (%), * compared with 2^nd^-line CAG group, † Fisher’s exact test, ‡ Log-rank test, § Mantel-Haenszel test, # compared with 2^nd^-line CAG patients with favorable and intermediate cytogenetics. (**D**) Event-free survival of the r/rAML patients from the start of r/rAML induction treatment to the date of relapse/refractory/death. (**E**) The statistical analysis of EFS of r/rAML patients at 2 years. Data are n/N (%), * compared with 2^nd^-line CAG group, † Fisher’s exact test, ‡ Log-rank test, § Mantel-Haenszel test, # compared with 2^nd^-line CAG patients with favorable and intermediate cytogenetics

A total of 186 r/rAML patients, treated between July 2012 and July 2022, were enrolled in the study. In this cohort, fit patients with *de novo* AML typically received Ida in combination with cytarabine (IA) for first-line therapy, as Ida is more effective than Doxo or Daun for AML induction therapy [51, 52]. However, all three anthracyclines are known to be cardiotoxic [53]. Among patients who developed refractory or relapse after primary IA treatment, 66 received CAG regimen (2^nd^-line CAG group), 67 received other chemotherapy regimens (including IA, DA, FLAG, CLAAG and CHA regimens, named collectively as 2^nd^-line others group), and 34 received emerging target therapy regimen venetoclax plus azacitidine (2^nd^-line VA group) (Fig. 5A). Notably, at Ruijin hospital, CAG is also applied to unfit *de novo* AML patients who are ineligible for conventional intensive chemotherapies. Therefore, 19 such patients treated with CAG for both *de novo* and r/r diseases were included to illustrate the lack of cardiotoxicity of Acla and its compatibility with extended treatment (1^st^&2^nd^-line CAG group) (Fig. 5A). The demographic and clinical characteristics of all patients at r/rAML diagnosis are summarized in Table S1. There was no significant difference between 2^nd^-line CAG group and any other group with respect to age, sex, French-American-British type, cytogenetic risk, or frequent oncogenic mutations. Slightly more patients in the 2^nd^-line others group were diagnosed with favorable integrated risk than in the other arms of the study.

The overall complete remission (CR) rate was 74% for the 2^nd^-line CAG group, significantly higher than that of the 2^nd^-line others (37%) and the 2^nd^-line VA group (35%) (Table 1). Patients with favorable and intermediate cytogenetics or integrated risk responded better to CAG, although these factors were not prognostic in the other groups (Table 1). As age being a poor prognostic factor for AML [47], in patients aged ≥ 60-year-old, 2^nd^-line CAG also outperformed other treatment regimens (Table S2). No CR and major prognostic mutation associations were detected in any treatment group due to the small cohort size (Table S2). Despite slightly less optimal outcomes for the 1^st^&2^nd^-line CAG group due to unfit conditions, this treatment protocol remained non-inferior to the 2^nd^-line others and the 2^nd^-line VA group (Table 1; Table S2), suggesting the continued effectiveness and well tolerability of CAG in unfit r/rAML patients, even after prior CAG treatments.

**Table 1:** Treatment outcomes of r/rAML patients

Clinical outcome	2^nd^-line CAG		2^nd^-line Others			2^nd^-line VA			1^st^&2^nd^-line CAG		
**Complete remission**	(*n* = 65)		(*n* = 51)		*P* value	(*n* = 31)		*P* value	(*n* = 17)		*P* value
Overall	48/65	(74%)	19/51	(37%)	**0.0001†**	11/31	(35%)	**0.0006†**	10/17	(59%)	0.2433†
**Complete remission**		**0.0279‡**			> 0.9999‡			> 0.9999‡			0.5500‡
Favorable and intermediate cytogenetics	40/50	(80%)	17/46	(37%)	**< 0.0001†**	8/23	(35%)	**0.0004†**	8/13	(62%)	0.2704†
Unfavorable cytogenetics	5/11	(45%)	2/5	(40%)	> 0.9999†	2/5	(40%)	> 0.9999†	1/3	(33%)	> 0.9999†
Unknown cytogenetics	3/4	(75%)	0/0	(NA)	> 0.9999†	1/2	(50%)	> 0.9999†	1/1	(100%)	> 0.9999†
**Complete remission**		**0.0087‡**			> 0.9999‡			0.2553‡			> 0.9999‡
Favorable and intermediate integrated risk	34/40	(85%)	12/32	(38%)	**< 0.0001†**	5/19	(26%)	**< 0.0001†**	6/10	(60%)	0.0966†
Unfavorable integrated risk	9/18	(50%)	6/16	(38%)	0.7055†	6/12	(50%)	> 0.9999†	3/5	(60%)	> 0.9999†
Unknown integrated risk	5/7	(71%)	1/3	(33%)	0.5000†	0/0	(NA)	> 0.9999†	1/2	(50%)	> 0.9999†

CAG: cytarabin, aclarubicin, G-CSF; IA: idarubicin, cytarabine; VA: venetoclax, azacitidine; Others: chemotherapies other than CAG, including IA, DA, FLAG, CLAAG and CHA regimens. Data are n/N (%). † Fisher’s exact test between the indicated group and 2^nd^-line CAG group. ‡ Fisher’s exact test within group

Given to the favorable tolerability of both CAG and VA therapy, some patients continued with these regimens for consolidation therapy and long-term maintenance, with some patients receiving up to 11 cycles of CAG without any cardiac issues, a far more cumulative dose than other anthracyclines (Fig. S5A). This extended treatment strategy was not an option for other anthracyclines due to accumulated cardiotoxicity and resistance. The superior CR rates and/or sustainable treatment schedules of CAG and VA translated into a more than 20% better OS compared to 2^nd^-line others group (Fig. 5B, C; Fig. S5B, C). However, event-free survival analysis indicated a more durable response with 2^nd^-line CAG compared to 2^nd^-line VA (Fig. 5D, E), possibly due to the development of acquired resistance to VA [54]. Of note, the 1^st^&2^nd^-line CAG patients assign to this low toxic CAG regimen because of unfit conditions, achieved comparable OS as the 2^nd^-line CAG group (Fig. 5B, C; Fig. S5D, E). Taken together, these results reveal the potential of CAG regimen as a superior, low-toxicity chemotherapy for r/rAML, including the unfit cases.

## Discussion

Anthracyclines like Doxo, Daun and Ida have been cornerstones of oncology treatment for over 50 years despite their devastating side effects. Contrary to the assumption of uniformity in molecular mechanisms across anthracycline analogs, our comprehensive analyses reveal substantial differences in mode of action, epigenomic selectivity, clinical performance, and pharmacological properties. In the course of our evaluation, we noted that Acla stands out for its potent anti-neoplastic effect, minimal cardiotoxicity and preferential distribution in lymphoid organs. The latter may explain the effectiveness of Acla in treating AML other than solid tumors. Attributing to its high therapeutic index for AML, we demonstrate that Acla can be safely used in second-line therapy following the treatment of cardiotoxic anthracyclines, leading to a 23% higher 5-year OS for r/rAML patients compared to other intensive chemotherapies.

DNA damage induced by TopoII poisoning has always been considered as the primary cytotoxic mechanism of anthracyclines [1]. However, this dogma is challenged by Acla and other anthracycline variants which predominantly elicit chromatin damage through histone eviction and TopoIIα redistribution [5, 55, 56]. TopoIIα plays roles in all areas of chromosome structure, including nucleosome turnover, chromosome condensation and segregation [57–59]. Moreover, it is exclusively expressed in proliferating compartments and is correlated with aggressive or rapidly proliferating cancers [60]. Deregulation of TopoIIα leads to cell death independent of its catalytic activity [61, 62]. Therefore, without poisoning TopoII for DNA damage, depleting and redistributing TopoIIα are cytotoxic, as observed with Acla, underscoring the significance of chromatin damage against cancer. Of note, the loss of TopoIIβ does not impede cell proliferation [63] and its expression does not influence the treatment outcome of anthracycline-based therapy in AML patients. Yet, it plays multifaceted role in transcription regulation [64] and contributes to therapy-related malignancies [65]. Hence, further exploration is warranted to delineate the effects of individual anthracyclines on TopoIIβ.

While in vitro and preclinical models allow for controlled investigations of single agent, they may not fully reflect the complexities of clinical scenarios. Single-center retrospective analyses also have inherent limitations, such as relative low sample size and confounding variables. Our dataset included the information on many features such as age, sex and mutations, revealing no discernible bias in any of the treatment arms. This allowed us to compare the treatment results between the 2^nd^-line CAG and other groups. The relative small size of 1^st^&2^nd^-line CAG group and shorter follow-up of 2^nd^-VA group may impact the statistical power. Notably, the focus of the 1^st^&2^nd^-line CAG group centers on its low toxicity and low acquired resistance, while venetoclax was introduced into clinic very recently.

While Acla was first introduced into clinic 40 years ago (it was available in Europe until 2004 and was never registered in the U.S.), the unique effectiveness of Acla in r/rAML has been overlooked. Besides the improvement in OS of r/rAML patients by CAG therapy, the preliminary results from our research group also suggest the feasibility and tolerability of CAG-VA combination (unpublished observations) warranting further investigation into its efficacy. By repurposing old drugs like Acla, an estimated 10,000 r/rAML patients annually in the U.S. and Europe [66] could survive from this off-patent and low cost drug. The impact on pediatric oncology will be even more profound, as 50% of childhood cancer patients receive high-dose anthracyclines and go on to have a 5- to 15-fold increased risk for heart failure compared to the general population [13]. Introducing Acla could significantly mitigate this risk [5, 56]. Considering old drugs in new ways may therefore substantially – and expeditiously – improve the survival and quality of life of many cancer patients, as exemplified here. Expanding on these findings, a multicenter Phase III prospective study is scheduled to commence later this year in China and the Netherlands, aiming to incorporate Acla into the treatment of r/rAML patients.

### Electronic supplementary material

Below is the link to the electronic supplementary material.

Supplementary Material 1

Supplementary Material 2

## Data Availability

The sequencing data supporting the findings of this study are available at the Gene Expression Omnibus (GEO) under accession numbers GSE240443 (https://www.ncbi.nlm.nih.gov/geo/query/acc.cgi?acc=GSE240443). Source data are provided with this paper. Clinical data are available from the corresponding authors upon reasonable request and with permission of Ruijin hospital.

## Abbreviations

Acla: Aclarubicin
AML: Acute myeloid leukemia
Amr: Amrubicin
Ara-C: Cytarabine
ATAC-seq: Assay for Transposase-Accessible Chromatin using sequencing
ATRA: Retinoic acid
CAG: Cytarabine, Acla and G-CSF regimen
CFGE: Constant-field gel electrophoresis
CHA: Cladribine, homoharringtonine and Ara-C regimen
ChIP-seq: Chromatin immunoprecipitation followed by sequencing
CLAAG: Cladribine, Ara-C, ATRA and G-CSF regimen
CR: Complete remission
DA: Decitabine and Ara-C regimen
Daun: Daunorubicin
DHS: DNase I hypersensitive regions
Doxo: Doxorubicin
Epi: Epirubicin
FCS: Fetal calf serum
FLAG: Fludarabine, Ara-C and G-CSF regimen
FLT3-ITD: FLT3 internal tandem duplication
G-CSF: Granulocyte colony stimulating factor
GEO: Gene expression omnibus
IA: Ida and cytarabine regimen
Ida: Idarubicin
IMDM: Iscove’s modified dulbecco’s medium
IR: Incidence rate
i.v.: Intravenous(ly)
OS: Overall survival
PCA: Principal component analysis
γH2AX: phosphorylation of H2AX at Ser139
r/rAML: relapsed/refractory AML
s.c.: Subcutaneous(ly)
SDS: Sodium dodecyl sulfate
TopoII: Topoisomerase II
VA: Venetoclax and azacitidine regimen
